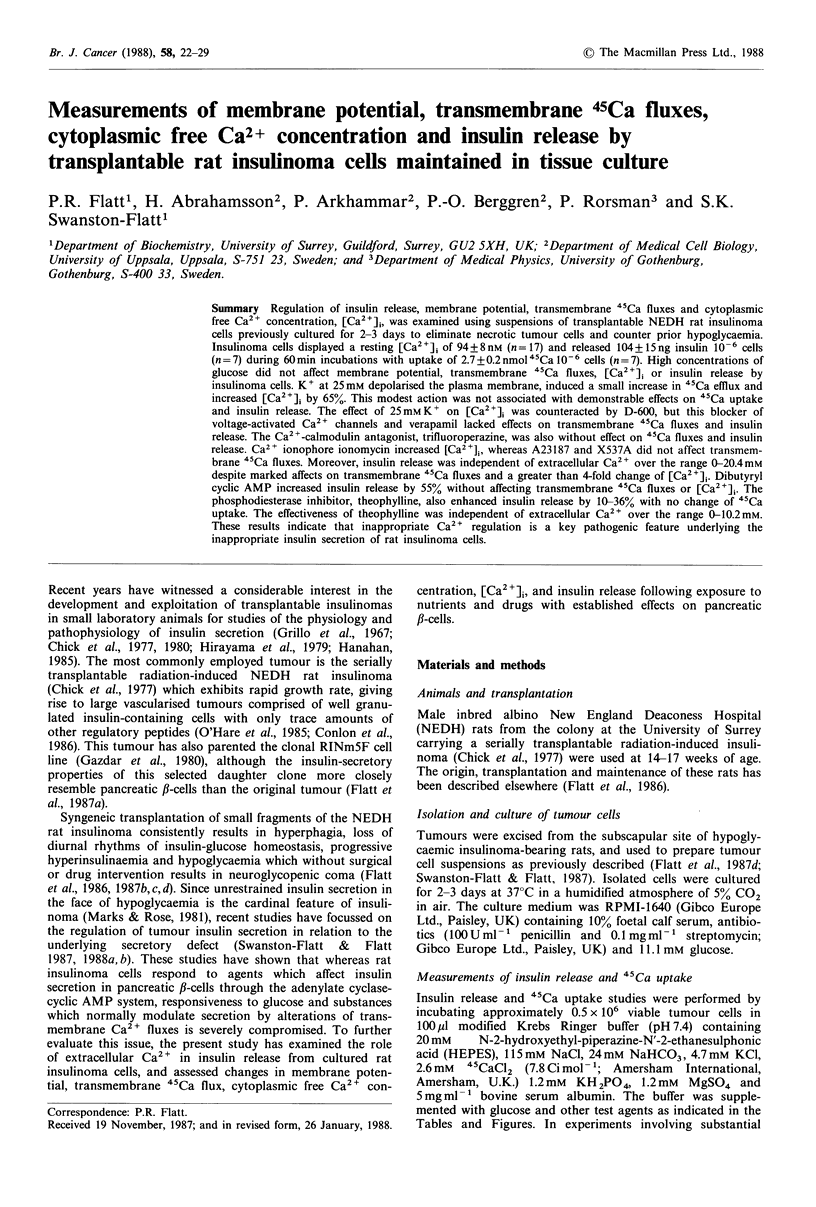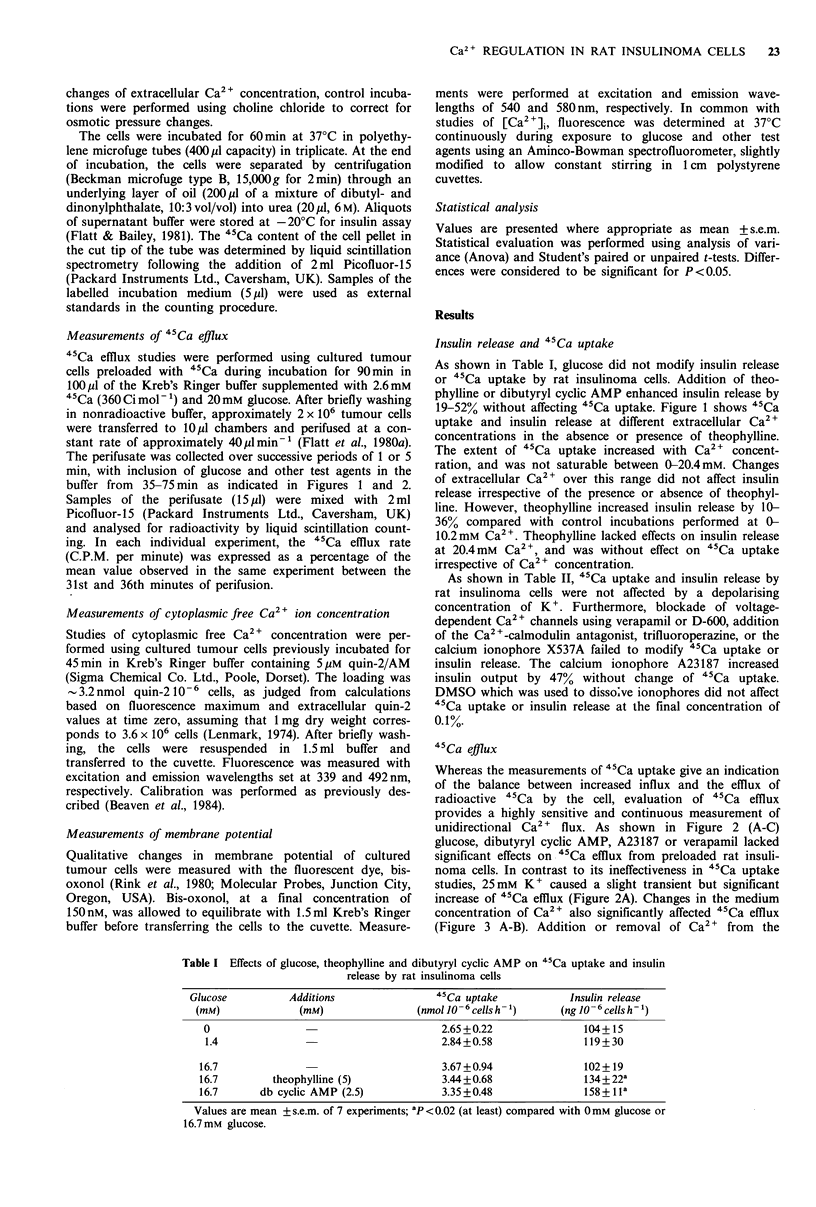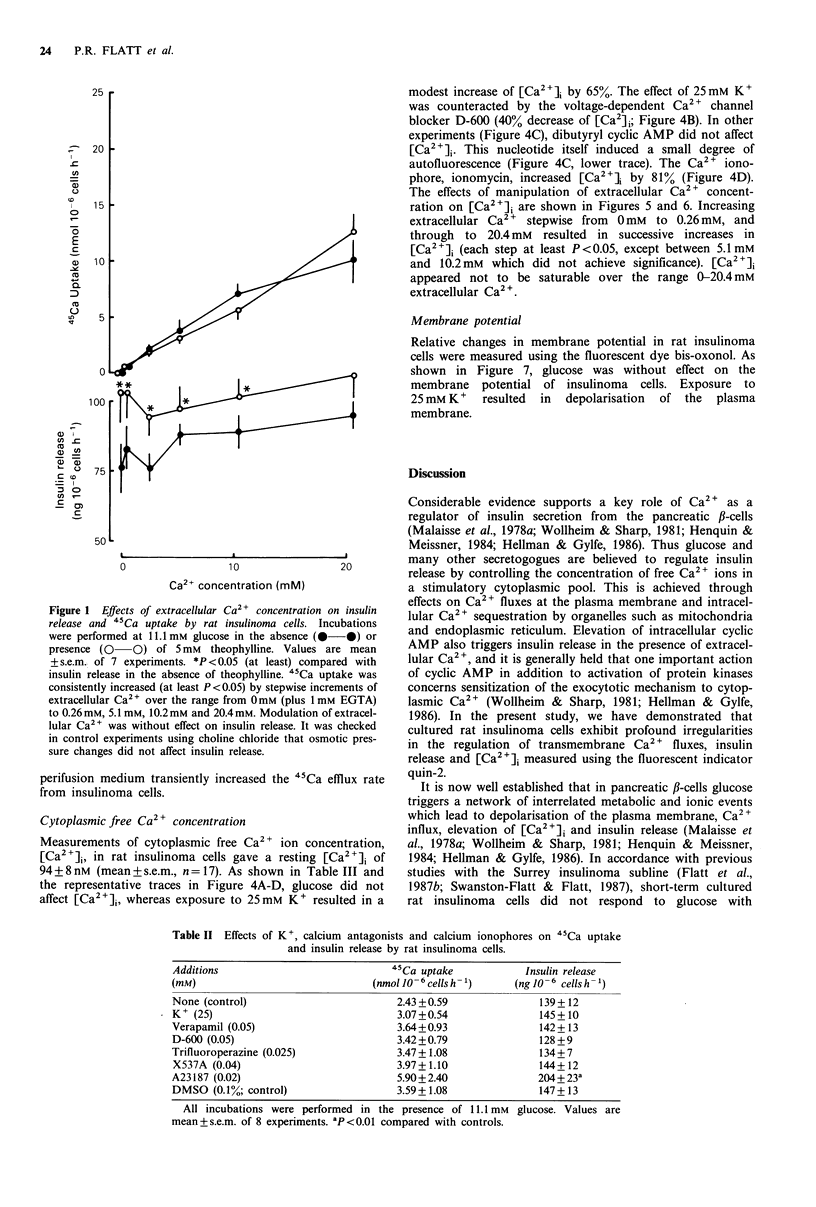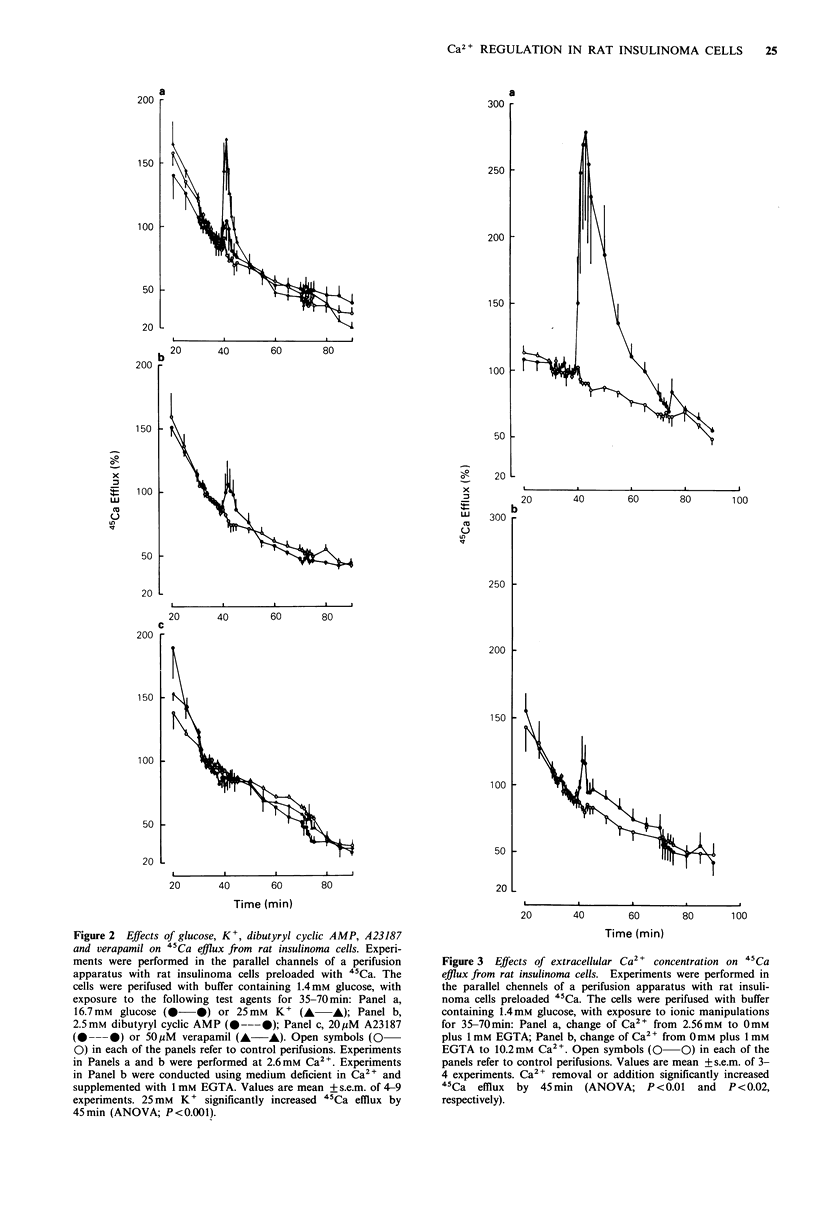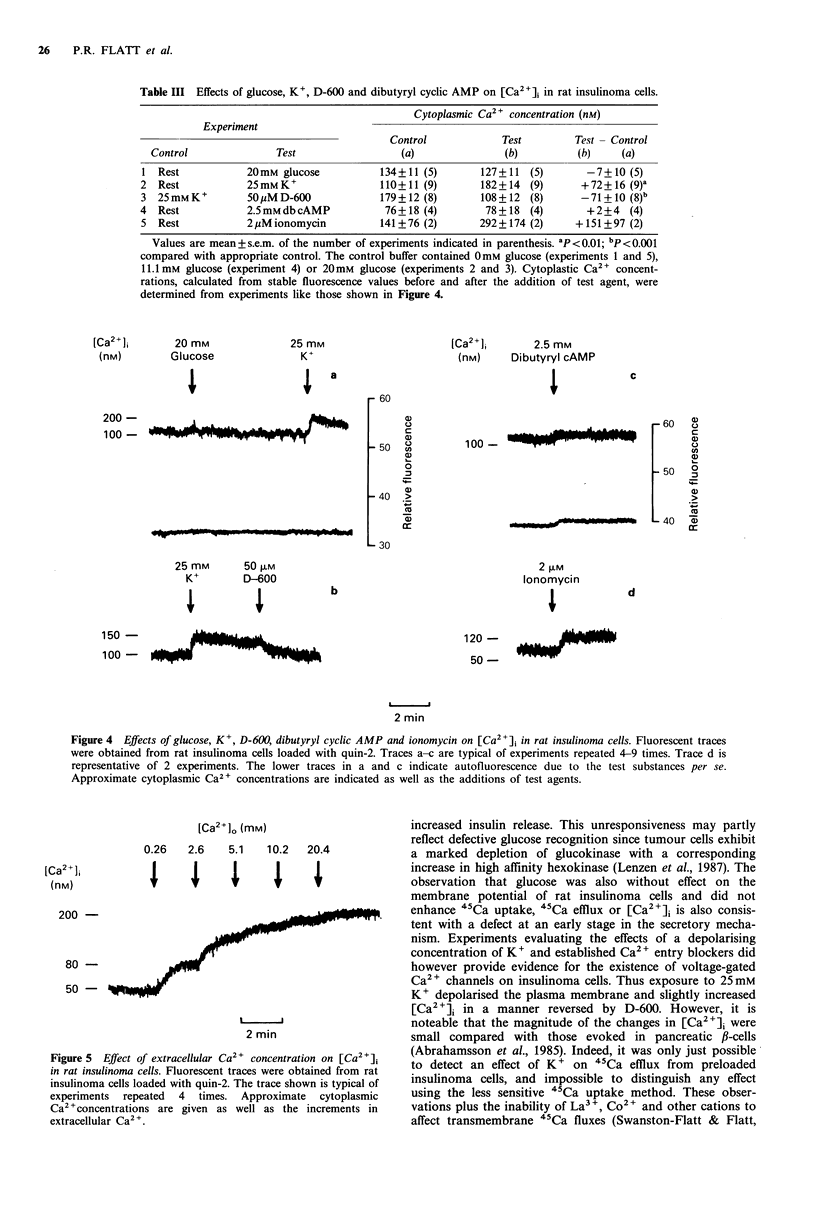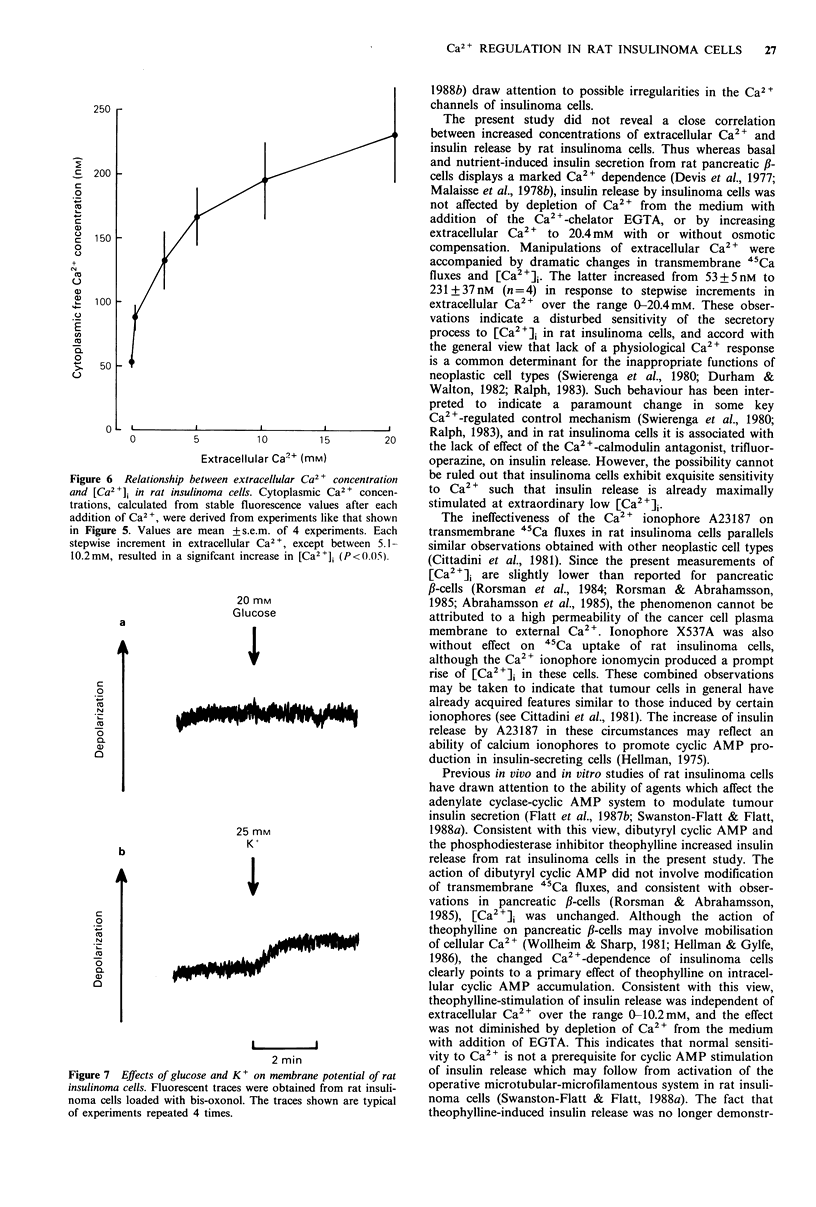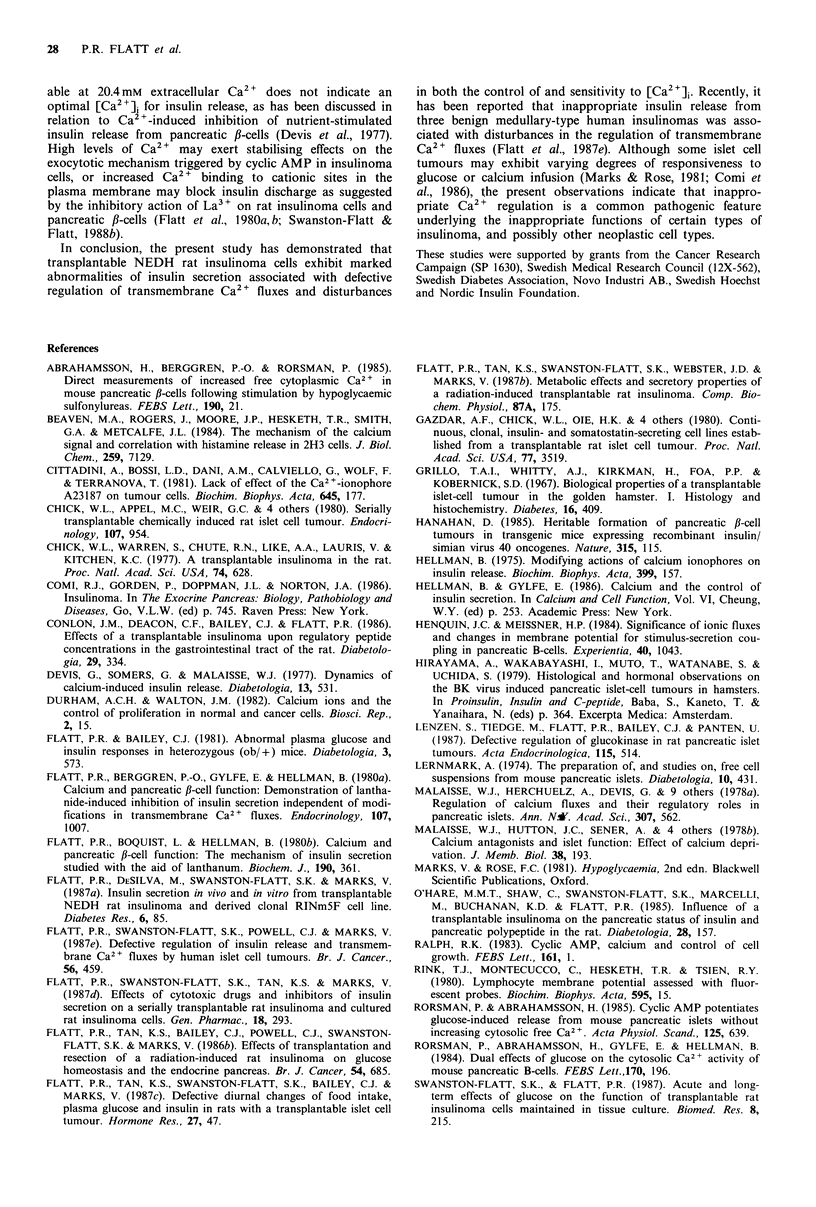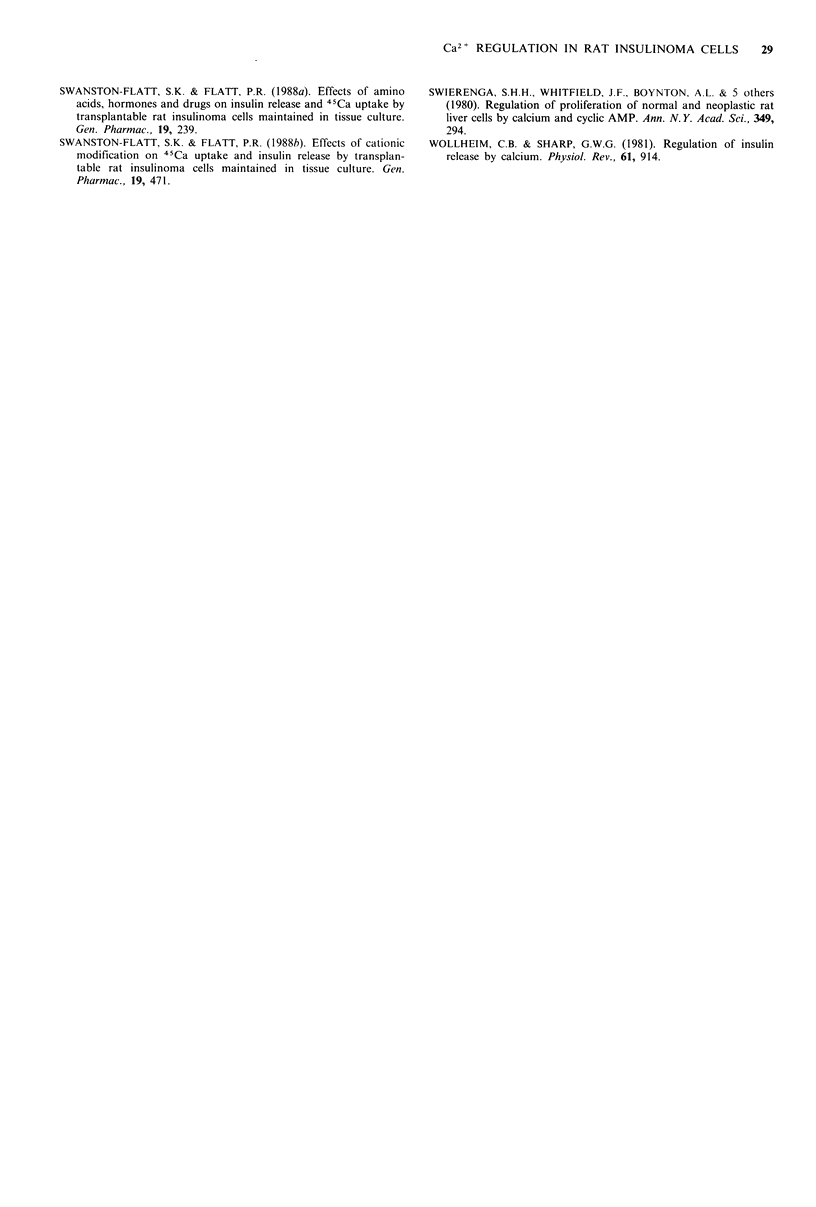# Measurements of membrane potential, transmembrane 45Ca fluxes, cytoplasmic free Ca2+ concentration and insulin release by transplantable rat insulinoma cells maintained in tissue culture.

**DOI:** 10.1038/bjc.1988.154

**Published:** 1988-07

**Authors:** P. R. Flatt, H. Abrahamsson, P. Arkhammar, P. O. Berggren, P. Rorsman, S. K. Swanston-Flatt

**Affiliations:** Department of Biochemistry, University of Surrey, Guildford, UK.

## Abstract

Regulation of insulin release, membrane potential, transmembrane 45Ca fluxes and cytoplasmic free Ca2+ concentration, [Ca2+]i, was examined using suspensions of transplantable NEDH rat insulinoma cells previously cultured for 2-3 days to eliminate necrotic tumour cells and counter prior hypoglycaemia. Insulinoma cells displayed a resting [Ca2+]i of 94 +/- 8 nM (n = 17) and released 104 +/- 15 ng insulin 10(-6) cells (n = 7) during 60 min incubations with uptake of 2.7 +/- 0.2 nmol 45Ca 10(-6) cells (n = 7). High concentrations of glucose did not affect membrane potential, transmembrane 45Ca fluxes, [Ca2+]i or insulin release by insulinoma cells. K+ at 25 mM depolarised the plasma membrane, induced a small increase in 45Ca efflux and increased [Ca2+]i by 65%. This modest action was not associated with demonstrable effects on 45Ca uptake and insulin release. The effect of 25 mMK+ on [Ca2+]i was counteracted by D-600, but this blocker of voltage-activated Ca2+ channels and verapamil lacked effects on transmembrane 45Ca fluxes and insulin release. The Ca2+-calmodulin antagonist, trifluoroperazine, was also without effect on 45Ca fluxes and insulin release. Ca2+ ionophore ionomycin increased [Ca2+]i, whereas A23187 and X537A did not affect transmembrane 45Ca fluxes. Moreover, insulin release was independent of extracellular Ca2+ over the range 0-20.4 mM despite marked affects on transmembrane 45Ca fluxes and a greater than 4-fold change of [Ca2+]i. Dibutyryl cyclic AMP increased insulin release by 55% without affecting transmembrane 45Ca fluxes or [Ca2+]i. The phosphodiesterase inhibitor, theophylline, also enhanced insulin release by 10-36% with no change of 45Ca uptake. The effectiveness of theophylline was independent of extracellular Ca2+ over the range 0-10.2 mM. These results indicate that inappropriate Ca2+ regulation is a key pathogenic feature underlying the inappropriate insulin secretion of rat insulinoma cells.


*Br. J. Cancer* (1988), **58**, 22–29

© The Macmillan Press Ltd., 1988

### Measurements of membrane potential, transmembrane $^{45}Ca$ fluxes, cytoplasmic free $Ca^{2+}$ concentration and insulin release by transplantable rat insulinoma cells maintained in tissue culture

P.R. Flatt[1], H. Abrahamsson[2], P. Arkhammar[2], P.-O. Berggren[2], P. Rorsman[3] and S.K. Swanston-Flatt[1]

[1]*Department of Biochemistry, University of Surrey, Guildford, Surrey, GU2 5XH, UK;* [2]*Department of Medical Cell Biology, University of Uppsala, Uppsala, S-751 23, Sweden; and* [3]*Department of Medical Physics, University of Gothenburg, Gothenburg, S-400 33, Sweden.*

**Summary** Regulation of insulin release, membrane potential, transmembrane $^{45}Ca$ fluxes and cytoplasmic free $Ca^{2+}$ concentration, $[Ca^{2+}]_i$, was examined using suspensions of transplantable NEDH rat insulinoma cells previously cultured for 2–3 days to eliminate necrotic tumour cells and counter prior hypoglycaemia. Insulinoma cells displayed a resting $[Ca^{2+}]_i$ of $94 \pm 8$ nM ($n=17$) and released $104 \pm 15$ ng insulin $10^{-6}$ cells ($n=7$) during 60 min incubations with uptake of $2.7 \pm 0.2$ nmol $^{45}Ca$ $10^{-6}$ cells ($n=7$). High concentrations of glucose did not affect membrane potential, transmembrane $^{45}Ca$ fluxes, $[Ca^{2+}]_i$ or insulin release by insulinoma cells. $K^+$ at 25 mM depolarised the plasma membrane, induced a small increase in $^{45}Ca$ efflux and increased $[Ca^{2+}]_i$ by 65%. This modest action was not associated with demonstrable effects on $^{45}Ca$ uptake and insulin release. The effect of 25 mM $K^+$ on $[Ca^{2+}]_i$ was counteracted by D-600, but this blocker of voltage-activated $Ca^{2+}$ channels and verapamil lacked effects on transmembrane $^{45}Ca$ fluxes and insulin release. The $Ca^{2+}$-calmodulin antagonist, trifluoroperazine, was also without effect on $^{45}Ca$ fluxes and insulin release. $Ca^{2+}$ ionophore ionomycin increased $[Ca^{2+}]_i$, whereas A23187 and X537A did not affect transmembrane $^{45}Ca$ fluxes. Moreover, insulin release was independent of extracellular $Ca^{2+}$ over the range 0–20.4 mM despite marked affects on transmembrane $^{45}Ca$ fluxes and a greater than 4-fold change of $[Ca^{2+}]_i$. Dibutyryl cyclic AMP increased insulin release by 55% without affecting transmembrane $^{45}Ca$ fluxes or $[Ca^{2+}]_i$. The phosphodiesterase inhibitor, theophylline, also enhanced insulin release by 10–36% with no change of $^{45}Ca$ uptake. The effectiveness of theophylline was independent of extracellular $Ca^{2+}$ over the range 0–10.2 mM. These results indicate that inappropriate $Ca^{2+}$ regulation is a key pathogenic feature underlying the inappropriate insulin secretion of rat insulinoma cells.

Recent years have witnessed a considerable interest in the development and exploitation of transplantable insulinomas in small laboratory animals for studies of the physiology and pathophysiology of insulin secretion (Grillo *et al.*, 1967; Chick *et al.*, 1977, 1980; Hirayama *et al.*, 1979; Hanahan, 1985). The most commonly employed tumour is the serially transplantable radiation-induced NEDH rat insulinoma (Chick *et al.*, 1977) which exhibits rapid growth rate, giving rise to large vascularised tumours comprised of well granulated insulin-containing cells with only trace amounts of other regulatory peptides (O'Hare *et al.*, 1985; Conlon *et al.*, 1986). This tumour has also parented the clonal RINm5F cell line (Gazdar *et al.*, 1980), although the insulin-secretory properties of this selected daughter clone more closely resemble pancreatic $\beta$-cells than the original tumour (Flatt *et al.*, 1987*a*).

Syngeneic transplantation of small fragments of the NEDH rat insulinoma consistently results in hyperphagia, loss of diurnal rhythms of insulin-glucose homeostasis, progressive hyperinsulinaemia and hypoglycaemia which without surgical or drug intervention results in neuroglycopenic coma (Flatt *et al.*, 1986, 1987*b*, *c*, *d*). Since unrestrained insulin secretion in the face of hypoglycaemia is the cardinal feature of insulinoma (Marks & Rose, 1981), recent studies have focussed on the regulation of tumour insulin secretion in relation to the underlying secretory defect (Swanston-Flatt & Flatt 1987, 1988*a*, *b*). These studies have shown that whereas rat insulinoma cells respond to agents which affect insulin secretion in pancreatic $\beta$-cells through the adenylate cyclase-cyclic AMP system, responsiveness to glucose and substances which normally modulate secretion by alterations of transmembrane $Ca^{2+}$ fluxes is severely compromised. To further evaluate this issue, the present study has examined the role of extracellular $Ca^{2+}$ in insulin release from cultured rat insulinoma cells, and assessed changes in membrane potential, transmembrane $^{45}Ca$ flux, cytoplasmic free $Ca^{2+}$ concentration, $[Ca^{2+}]_i$, and insulin release following exposure to nutrients and drugs with established effects on pancreatic $\beta$-cells.

Correspondence: P.R. Flatt.

Received 19 November, 1987; and in revised form, 26 January, 1988.

### Materials and methods

### *Animals and transplantation*

Male inbred albino New England Deaconess Hospital (NEDH) rats from the colony at the University of Surrey carrying a serially transplantable radiation-induced insulinoma (Chick *et al.*, 1977) were used at 14–17 weeks of age. The origin, transplantation and maintenance of these rats has been described elsewhere (Flatt *et al.*, 1986).

### *Isolation and culture of tumour cells*

Tumours were excised from the subscapular site of hypoglycaemic insulinoma-bearing rats, and used to prepare tumour cell suspensions as previously described (Flatt *et al.*, 1987*d*; Swanston-Flatt & Flatt, 1987). Isolated cells were cultured for 2–3 days at 37°C in a humidified atmosphere of 5% $CO_2$ in air. The culture medium was RPMI-1640 (Gibco Europe Ltd., Paisley, UK) containing 10% foetal calf serum, antibiotics (100 U ml$^{-1}$ penicillin and 0.1 mg ml$^{-1}$ streptomycin; Gibco Europe Ltd., Paisley, UK) and 11.1 mM glucose.

### *Measurements of insulin release and $^{45}Ca$ uptake*

Insulin release and $^{45}Ca$ uptake studies were performed by incubating approximately $0.5 \times 10^6$ viable tumour cells in 100 $\mu$l modified Krebs Ringer buffer (pH 7.4) containing 20 mM N-2-hydroxyethyl-piperazine-N'-2-ethanesulphonic acid (HEPES), 115 mM NaCl, 24 mM $NaHCO_3$, 4.7 mM KCl, 2.6 mM $^{45}CaCl_2$ (7.8 Ci mol$^{-1}$; Amersham International, Amersham, U.K.) 1.2 mM $KH_2PO_4$, 1.2 mM $MgSO_4$ and 5 mg ml$^{-1}$ bovine serum albumin. The buffer was supplemented with glucose and other test agents as indicated in the Tables and Figures. In experiments involving substantial changes of extracellular $Ca^{2+}$ concentration, control incubations were performed using choline chloride to correct for osmotic pressure changes.

The cells were incubated for 60 min at 37°C in polyethylene microfuge tubes (400 µl capacity) in triplicate. At the end of incubation, the cells were separated by centrifugation (Beckman microfuge type B, 15,000 *g* for 2 min) through an underlying layer of oil (200 µl of a mixture of dibutyl- and dinonylphthalate, 10:3 vol/vol) into urea (20 µl, 6 M). Aliquots of supernatant buffer were stored at −20°C for insulin assay (Flatt & Bailey, 1981). The $^{45}Ca$ content of the cell pellet in the cut tip of the tube was determined by liquid scintillation spectrometry following the addition of 2 ml Picofluor-15 (Packard Instruments Ltd., Caversham, UK). Samples of the labelled incubation medium (5 µl) were used as external standards in the counting procedure.

*Measurements of $^{45}Ca$ efflux*

$^{45}Ca$ efflux studies were performed using cultured tumour cells preloaded with $^{45}Ca$ during incubation for 90 min in 100 µl of the Kreb's Ringer buffer supplemented with 2.6 mM $^{45}Ca$ (360 Ci mol$^{-1}$) and 20 mM glucose. After briefly washing in nonradioactive buffer, approximately $2 \times 10^6$ tumour cells were transferred to 10 µl chambers and perifused at a constant rate of approximately 40 µl min$^{-1}$ (Flatt *et al.*, 1980*a*). The perifusate was collected over successive periods of 1 or 5 min, with inclusion of glucose and other test agents in the buffer from 35–75 min as indicated in Figures 1 and 2. Samples of the perifusate (15 µl) were mixed with 2 ml Picofluor-15 (Packard Instruments Ltd., Caversham, UK) and analysed for radioactivity by liquid scintillation counting. In each individual experiment, the $^{45}Ca$ efflux rate (C.P.M. per minute) was expressed as a percentage of the mean value observed in the same experiment between the 31st and 36th minutes of perifusion.

*Measurements of cytoplasmic free $Ca^{2+}$ ion concentration*

Studies of cytoplasmic free $Ca^{2+}$ concentration were performed using cultured tumour cells previously incubated for 45 min in Kreb's Ringer buffer containing 5 µM quin-2/AM (Sigma Chemical Co. Ltd., Poole, Dorset). The loading was ~3.2 nmol quin-2 $10^{-6}$ cells, as judged from calculations based on fluorescence maximum and extracellular quin-2 values at time zero, assuming that 1 mg dry weight corresponds to $3.6 \times 10^6$ cells (Lenmark, 1974). After briefly washing, the cells were resuspended in 1.5 ml buffer and transferred to the cuvette. Fluorescence was measured with excitation and emission wavelengths set at 339 and 492 nm, respectively. Calibration was performed as previously described (Beaven *et al.*, 1984).

*Measurements of membrane potential*

Qualitative changes in membrane potential of cultured tumour cells were measured with the fluorescent dye, bis-oxonol (Rink *et al.*, 1980; Molecular Probes, Junction City, Oregon, USA). Bis-oxonol, at a final concentration of 150 nM, was allowed to equilibrate with 1.5 ml Kreb's Ringer buffer before transferring the cells to the cuvette. Measurements were performed at excitation and emission wavelengths of 540 and 580 nm, respectively. In common with studies of $[Ca^{2+}]_i$, fluorescence was determined at 37°C continuously during exposure to glucose and other test agents using an Aminco-Bowman spectrofluorometer, slightly modified to allow constant stirring in 1 cm polystyrene cuvettes.

*Statistical analysis*

Values are presented where appropriate as mean ±s.e.m. Statistical evaluation was performed using analysis of variance (Anova) and Student's paired or unpaired *t*-tests. Differences were considered to be significant for $P < 0.05$.

### Results

*Insulin release and $^{45}Ca$ uptake*

As shown in Table I, glucose did not modify insulin release or $^{45}Ca$ uptake by rat insulinoma cells. Addition of theophylline or dibutyryl cyclic AMP enhanced insulin release by 19–52% without affecting $^{45}Ca$ uptake. Figure 1 shows $^{45}Ca$ uptake and insulin release at different extracellular $Ca^{2+}$ concentrations in the absence or presence of theophylline. The extent of $^{45}Ca$ uptake increased with $Ca^{2+}$ concentration, and was not saturable between 0–20.4 mM. Changes of extracellular $Ca^{2+}$ over this range did not affect insulin release irrespective of the presence or absence of theophylline. However, theophylline increased insulin release by 10–36% compared with control incubations performed at 0–10.2 mM $Ca^{2+}$. Theophylline lacked effects on insulin release at 20.4 mM $Ca^{2+}$, and was without effect on $^{45}Ca$ uptake irrespective of $Ca^{2+}$ concentration.

As shown in Table II, $^{45}Ca$ uptake and insulin release by rat insulinoma cells were not affected by a depolarising concentration of $K^+$. Furthermore, blockade of voltage-dependent $Ca^{2+}$ channels using verapamil or D-600, addition of the $Ca^{2+}$-calmodulin antagonist, trifluoroperazine, or the calcium ionophore X537A failed to modify $^{45}Ca$ uptake or insulin release. The calcium ionophore A23187 increased insulin output by 47% without change of $^{45}Ca$ uptake. DMSO which was used to dissolve ionophores did not affect $^{45}Ca$ uptake or insulin release at the final concentration of 0.1%.

*$^{45}Ca$ efflux*

Whereas the measurements of $^{45}Ca$ uptake give an indication of the balance between increased influx and the efflux of radioactive $^{45}Ca$ by the cell, evaluation of $^{45}Ca$ efflux provides a highly sensitive and continuous measurement of unidirectional $Ca^{2+}$ flux. As shown in Figure 2 (A-C) glucose, dibutyryl cyclic AMP, A23187 or verapamil lacked significant effects on $^{45}Ca$ efflux from preloaded rat insulinoma cells. In contrast to its ineffectiveness in $^{45}Ca$ uptake studies, 25 mM $K^+$ caused a slight transient but significant increase of $^{45}Ca$ efflux (Figure 2A). Changes in the medium concentration of $Ca^{2+}$ also significantly affected $^{45}Ca$ efflux (Figure 3 A-B). Addition or removal of $Ca^{2+}$ from the

**Table I** Effects of glucose, theophylline and dibutyryl cyclic AMP on $^{45}Ca$ uptake and insulin release by rat insulinoma cells

| *Glucose* (*mM*) | *Additions* (*mM*) | *$^{45}Ca$ uptake* (*nmol $10^{-6}$ cells $h^{-1}$*) | *Insulin release* (*ng $10^{-6}$ cells $h^{-1}$*) |
|---|---|---|---|
| 0 | — | 2.65 ± 0.22 | 104 ± 15 |
| 1.4 | — | 2.84 ± 0.58 | 119 ± 30 |
| 16.7 | — | 3.67 ± 0.94 | 102 ± 19 |
| 16.7 | theophylline (5) | 3.44 ± 0.68 | 134 ± 22[a] |
| 16.7 | db cyclic AMP (2.5) | 3.35 ± 0.48 | 158 ± 11[a] |

Values are mean ±s.e.m. of 7 experiments; [a]$P < 0.02$ (at least) compared with 0 mM glucose or 16.7 mM glucose.

**Figure 1** *Effects of extracellular $Ca^{2+}$ concentration on insulin release and $^{45}Ca$ uptake by rat insulinoma cells.* Incubations were performed at 11.1 mM glucose in the absence (●——●) or presence (○——○) of 5 mM theophylline. Values are mean ± s.e.m. of 7 experiments. *$P<0.05$ (at least) compared with insulin release in the absence of theophylline. $^{45}$Ca uptake was consistently increased (at least $P<0.05$) by stepwise increments of extracellular $Ca^{2+}$ over the range from 0 mM (plus 1 mM EGTA) to 0.26 mM, 5.1 mM, 10.2 mM and 20.4 mM. Modulation of extracellular $Ca^{2+}$ was without effect on insulin release. It was checked in control experiments using choline chloride that osmotic pressure changes did not affect insulin release.

perifusion medium transiently increased the $^{45}$Ca efflux rate from insulinoma cells.

*Cytoplasmic free $Ca^{2+}$ concentration*

Measurements of cytoplasmic free $Ca^{2+}$ ion concentration, $[Ca^{2+}]_i$, in rat insulinoma cells gave a resting $[Ca^{2+}]_i$ of 94±8 nM (mean±s.e.m., $n=17$). As shown in Table III and the representative traces in Figure 4A-D, glucose did not affect $[Ca^{2+}]_i$, whereas exposure to 25 mM $K^+$ resulted in a modest increase of $[Ca^{2+}]_i$ by 65%. The effect of 25 mM $K^+$ was counteracted by the voltage-dependent $Ca^{2+}$ channel blocker D-600 (40% decrease of $[Ca^2]_i$; Figure 4B). In other experiments (Figure 4C), dibutyryl cyclic AMP did not affect $[Ca^{2+}]_i$. This nucleotide itself induced a small degree of autofluorescence (Figure 4C, lower trace). The $Ca^{2+}$ ionophore, ionomycin, increased $[Ca^{2+}]_i$ by 81% (Figure 4D). The effects of manipulation of extracellular $Ca^{2+}$ concentration on $[Ca^{2+}]_i$ are shown in Figures 5 and 6. Increasing extracellular $Ca^{2+}$ stepwise from 0 mM to 0.26 mM, and through to 20.4 mM resulted in successive increases in $[Ca^{2+}]_i$ (each step at least $P<0.05$, except between 5.1 mM and 10.2 mM which did not achieve significance). $[Ca^{2+}]_i$ appeared not to be saturable over the range 0–20.4 mM extracellular $Ca^{2+}$.

*Membrane potential*

Relative changes in membrane potential in rat insulinoma cells were measured using the fluorescent dye bis-oxonol. As shown in Figure 7, glucose was without effect on the membrane potential of insulinoma cells. Exposure to 25 mM $K^+$ resulted in depolarisation of the plasma membrane.

### Discussion

Considerable evidence supports a key role of $Ca^{2+}$ as a regulator of insulin secretion from the pancreatic β-cells (Malaisse *et al.*, 1978*a*; Wollheim & Sharp, 1981; Henquin & Meissner, 1984; Hellman & Gylfe, 1986). Thus glucose and many other secretogogues are believed to regulate insulin release by controlling the concentration of free $Ca^{2+}$ ions in a stimulatory cytoplasmic pool. This is achieved through effects on $Ca^{2+}$ fluxes at the plasma membrane and intracellular $Ca^{2+}$ sequestration by organelles such as mitochondria and endoplasmic reticulum. Elevation of intracellular cyclic AMP also triggers insulin release in the presence of extracellular $Ca^{2+}$, and it is generally held that one important action of cyclic AMP in addition to activation of protein kinases concerns sensitization of the exocytotic mechanism to cytoplasmic $Ca^{2+}$ (Wollheim & Sharp, 1981; Hellman & Gylfe, 1986). In the present study, we have demonstrated that cultured rat insulinoma cells exhibit profound irregularities in the regulation of transmembrane $Ca^{2+}$ fluxes, insulin release and $[Ca^{2+}]_i$ measured using the fluorescent indicator quin-2.

It is now well established that in pancreatic β-cells glucose triggers a network of interrelated metabolic and ionic events which lead to depolarisation of the plasma membrane, $Ca^{2+}$ influx, elevation of $[Ca^{2+}]_i$ and insulin release (Malaisse *et al.*, 1978*a*; Wollheim & Sharp, 1981; Henquin & Meissner, 1984; Hellman & Gylfe, 1986). In accordance with previous studies with the Surrey insulinoma subline (Flatt *et al.*, 1987*b*; Swanston-Flatt & Flatt, 1987), short-term cultured rat insulinoma cells did not respond to glucose with

**Table II** Effects of $K^+$, calcium antagonists and calcium ionophores on $^{45}$Ca uptake and insulin release by rat insulinoma cells.

| *Additions* (*mM*) | *$^{45}$Ca uptake* (*nmol $10^{-6}$ cells $h^{-1}$*) | *Insulin release* (*ng $10^{-6}$ cells $h^{-1}$*) |
|---|---|---|
| None (control) | 2.43±0.59 | 139±12 |
| $K^+$ (25) | 3.07±0.54 | 145±10 |
| Verapamil (0.05) | 3.64±0.93 | 142±13 |
| D-600 (0.05) | 3.42±0.79 | 128±9 |
| Trifluoroperazine (0.025) | 3.47±1.08 | 134±7 |
| X537A (0.04) | 3.97±1.10 | 144±12 |
| A23187 (0.02) | 5.90±2.40 | 204±23[a] |
| DMSO (0.1%; control) | 3.59±1.08 | 147±13 |

All incubations were performed in the presence of 11.1 mM glucose. Values are mean±s.e.m. of 8 experiments. [a]$P<0.01$ compared with controls.

**Figure 2** *Effects of glucose, $K^+$, dibutyryl cyclic AMP, A23187 and verapamil on $^{45}Ca$ efflux from rat insulinoma cells.* Experiments were performed in the parallel channels of a perifusion apparatus with rat insulinoma cells preloaded with $^{45}$Ca. The cells were perifused with buffer containing 1.4 mM glucose, with exposure to the following test agents for 35–70 min: Panel a, 16.7 mM glucose (●——●) or 25 mM $K^+$ (▲——▲); Panel b, 2.5 mM dibutyryl cyclic AMP (●---●); Panel c, 20 $\mu$M A23187 (●---●) or 50 $\mu$M verapamil (▲——▲). Open symbols (○——○) in each of the panels refer to control perifusions. Experiments in Panels a and b were performed at 2.6 mM $Ca^{2+}$. Experiments in Panel b were conducted using medium deficient in $Ca^{2+}$ and supplemented with 1 mM EGTA. Values are mean ± s.e.m. of 4–9 experiments. 25 mM $K^+$ significantly increased $^{45}$Ca efflux by 45 min (ANOVA; $P<0.001$).

**Figure 3** *Effects of extracellular $Ca^{2+}$ concentration on $^{45}Ca$ efflux from rat insulinoma cells.* Experiments were performed in the parallel chennels of a perifusion apparatus with rat insulinoma cells preloaded $^{45}$Ca. The cells were perifused with buffer containing 1.4 mM glucose, with exposure to ionic manipulations for 35–70 min: Panel a, change of $Ca^{2+}$ from 2.56 mM to 0 mM plus 1 mM EGTA; Panel b, change of $Ca^{2+}$ from 0 mM plus 1 mM EGTA to 10.2 mM $Ca^{2+}$. Open symbols (○——○) in each of the panels refer to control perifusions. Values are mean ± s.e.m. of 3–4 experiments. $Ca^{2+}$ removal or addition significantly increased $^{45}$Ca efflux by 45 min (ANOVA; $P<0.01$ and $P<0.02$, respectively).

**Table III** Effects of glucose, $K^+$, D-600 and dibutyryl cyclic AMP on $[Ca^{2+}]_i$ in rat insulinoma cells.

| Experiment | | Cytoplasmic $Ca^{2+}$ concentration (nM) | | |
|---|---|---|---|---|
| Control | Test | Control (a) | Test (b) | Test − Control (b) (a) |
| 1 Rest | 20 mM glucose | 134±11 (5) | 127±11 (5) | −7±10 (5) |
| 2 Rest | 25 mM $K^+$ | 110±11 (9) | 182±14 (9) | +72±16 (9)[a] |
| 3 25 mM $K^+$ | 50 μM D-600 | 179±12 (8) | 108±12 (8) | −71±10 (8)[b] |
| 4 Rest | 2.5 mM db cAMP | 76±18 (4) | 78±18 (4) | +2±4 (4) |
| 5 Rest | 2 μM ionomycin | 141±76 (2) | 292±174 (2) | +151±97 (2) |

Values are mean ± s.e.m. of the number of experiments indicated in parenthesis. [a]$P<0.01$; [b]$P<0.001$ compared with appropriate control. The control buffer contained 0 mM glucose (experiments 1 and 5), 11.1 mM glucose (experiment 4) or 20 mM glucose (experiments 2 and 3). Cytoplastic $Ca^{2+}$ concentrations, calculated from stable fluorescence values before and after the addition of test agent, were determined from experiments like those shown in **Figure 4**.

**Figure 4** *Effects of glucose, $K^+$, D-600, dibutyryl cyclic AMP and ionomycin on $[Ca^{2+}]_i$ in rat insulinoma cells.* Fluorescent traces were obtained from rat insulinoma cells loaded with quin-2. Traces a–c are typical of experiments repeated 4–9 times. Trace d is representative of 2 experiments. The lower traces in a and c indicate autofluorescence due to the test substances *per se*. Approximate cytoplasmic $Ca^{2+}$ concentrations are indicated as well as the additions of test agents.

**Figure 5** *Effect of extracellular $Ca^{2+}$ concentration on $[Ca^{2+}]_i$ in rat insulinoma cells.* Fluorescent traces were obtained from rat insulinoma cells loaded with quin-2. The trace shown is typical of experiments repeated 4 times. Approximate cytoplasmic $Ca^{2+}$concentrations are given as well as the increments in extracellular $Ca^{2+}$.

increased insulin release. This unresponsiveness may partly reflect defective glucose recognition since tumour cells exhibit a marked depletion of glucokinase with a corresponding increase in high affinity hexokinase (Lenzen *et al.*, 1987). The observation that glucose was also without effect on the membrane potential of rat insulinoma cells and did not enhance $^{45}Ca$ uptake, $^{45}Ca$ efflux or $[Ca^{2+}]_i$ is also consistent with a defect at an early stage in the secretory mechanism. Experiments evaluating the effects of a depolarising concentration of $K^+$ and established $Ca^{2+}$ entry blockers did however provide evidence for the existence of voltage-gated $Ca^{2+}$ channels on insulinoma cells. Thus exposure to 25 mM $K^+$ depolarised the plasma membrane and slightly increased $[Ca^{2+}]_i$ in a manner reversed by D-600. However, it is noteable that the magnitude of the changes in $[Ca^{2+}]_i$ were small compared with those evoked in pancreatic β-cells (Abrahamsson *et al.*, 1985). Indeed, it was only just possible to detect an effect of $K^+$ on $^{45}Ca$ efflux from preloaded insulinoma cells, and impossible to distinguish any effect using the less sensitive $^{45}Ca$ uptake method. These observations plus the inability of $La^{3+}$, $Co^{2+}$ and other cations to affect transmembrane $^{45}Ca$ fluxes (Swanston-Flatt & Flatt,

**Figure 6** *Relationship between extracellular $Ca^{2+}$ concentration and $[Ca^{2+}]_i$ in rat insulinoma cells.* Cytoplasmic $Ca^{2+}$ concentrations, calculated from stable fluorescence values after each addition of $Ca^{2+}$, were derived from experiments like that shown in **Figure 5**. Values are mean ±s.e.m. of 4 experiments. Each stepwise increment in extracellular $Ca^{2+}$, except between 5.1–10.2 mM, resulted in a signifcant increase in $[Ca^{2+}]_i$ ($P<0.05$).

**Figure 7** *Effects of glucose and $K^+$ on membrane potential of rat insulinoma cells.* Fluorescent traces were obtained from rat insulinoma cells loaded with bis-oxonol. The traces shown are typical of experiments repeated 4 times.

1988*b*) draw attention to possible irregularities in the $Ca^{2+}$ channels of insulinoma cells.

The present study did not reveal a close correlation between increased concentrations of extracellular $Ca^{2+}$ and insulin release by rat insulinoma cells. Thus whereas basal and nutrient-induced insulin secretion from rat pancreatic $\beta$-cells displays a marked $Ca^{2+}$ dependence (Devis *et al.*, 1977; Malaisse *et al.*, 1978*b*), insulin release by insulinoma cells was not affected by depletion of $Ca^{2+}$ from the medium with addition of the $Ca^{2+}$-chelator EGTA, or by increasing extracellular $Ca^{2+}$ to 20.4 mM with or without osmotic compensation. Manipulations of extracellular $Ca^{2+}$ were accompanied by dramatic changes in transmembrane $^{45}Ca$ fluxes and $[Ca^{2+}]_i$. The latter increased from 53±5 nM to 231±37 nM ($n=4$) in response to stepwise increments in extracellular $Ca^{2+}$ over the range 0–20.4 mM. These observations indicate a disturbed sensitivity of the secretory process to $[Ca^{2+}]_i$ in rat insulinoma cells, and accord with the general view that lack of a physiological $Ca^{2+}$ response is a common determinant for the inappropriate functions of neoplastic cell types (Swierenga *et al.*, 1980; Durham & Walton, 1982; Ralph, 1983). Such behaviour has been interpreted to indicate a paramount change in some key $Ca^{2+}$-regulated control mechanism (Swierenga *et al.*, 1980; Ralph, 1983), and in rat insulinoma cells it is associated with the lack of effect of the $Ca^{2+}$-calmodulin antagonist, trifluoroperazine, on insulin release. However, the possibility cannot be ruled out that insulinoma cells exhibit exquisite sensitivity to $Ca^{2+}$ such that insulin release is already maximally stimulated at extraordinary low $[Ca^{2+}]_i$.

The ineffectiveness of the $Ca^{2+}$ ionophore A23187 on transmembrane $^{45}Ca$ fluxes in rat insulinoma cells parallels similar observations obtained with other neoplastic cell types (Cittadini *et al.*, 1981). Since the present measurements of $[Ca^{2+}]_i$ are slightly lower than reported for pancreatic $\beta$-cells (Rorsman *et al.*, 1984; Rorsman & Abrahamsson, 1985; Abrahamsson *et al.*, 1985), the phenomenon cannot be attributed to a high permeability of the cancer cell plasma membrane to external $Ca^{2+}$. Ionophore X537A was also without effect on $^{45}Ca$ uptake of rat insulinoma cells, although the $Ca^{2+}$ ionophore ionomycin produced a prompt rise of $[Ca^{2+}]_i$ in these cells. These combined observations may be taken to indicate that tumour cells in general have already acquired features similar to those induced by certain ionophores (see Cittadini *et al.*, 1981). The increase of insulin release by A23187 in these circumstances may reflect an ability of calcium ionophores to promote cyclic AMP production in insulin-secreting cells (Hellman, 1975).

Previous *in vivo* and *in vitro* studies of rat insulinoma cells have drawn attention to the ability of agents which affect the adenylate cyclase-cyclic AMP system to modulate tumour insulin secretion (Flatt *et al.*, 1987*b*; Swanston-Flatt & Flatt, 1988*a*). Consistent with this view, dibutyryl cyclic AMP and the phosphodiesterase inhibitor theophylline increased insulin release from rat insulinoma cells in the present study. The action of dibutyryl cyclic AMP did not involve modification of transmembrane $^{45}Ca$ fluxes, and consistent with observations in pancreatic $\beta$-cells (Rorsman & Abrahamsson, 1985), $[Ca^{2+}]_i$ was unchanged. Although the action of theophylline on pancreatic $\beta$-cells may involve mobilisation of cellular $Ca^{2+}$ (Wollheim & Sharp, 1981; Hellman & Gylfe, 1986), the changed $Ca^{2+}$-dependence of insulinoma cells clearly points to a primary effect of theophylline on intracellular cyclic AMP accumulation. Consistent with this view, theophylline-stimulation of insulin release was independent of extracellular $Ca^{2+}$ over the range 0–10.2 mM, and the effect was not diminished by depletion of $Ca^{2+}$ from the medium with addition of EGTA. This indicates that normal sensitivity to $Ca^{2+}$ is not a prerequisite for cyclic AMP stimulation of insulin release which may follow from activation of the operative microtubular-microfilamentous system in rat insulinoma cells (Swanston-Flatt & Flatt, 1988*a*). The fact that theophylline-induced insulin release was no longer demonstrable at 20.4 mM extracellular $Ca^{2+}$ does not indicate an optimal $[Ca^{2+}]_i$ for insulin release, as has been discussed in relation to $Ca^{2+}$-induced inhibition of nutrient-stimulated insulin release from pancreatic $\beta$-cells (Devis *et al.*, 1977). High levels of $Ca^{2+}$ may exert stabilising effects on the exocytotic mechanism triggered by cyclic AMP in insulinoma cells, or increased $Ca^{2+}$ binding to cationic sites in the plasma membrane may block insulin discharge as suggested by the inhibitory action of $La^{3+}$ on rat insulinoma cells and pancreatic $\beta$-cells (Flatt *et al.*, 1980*a,b*; Swanston-Flatt & Flatt, 1988*b*).

In conclusion, the present study has demonstrated that transplantable NEDH rat insulinoma cells exhibit marked abnormalities of insulin secretion associated with defective regulation of transmembrane $Ca^{2+}$ fluxes and disturbances in both the control of and sensitivity to $[Ca^{2+}]_i$. Recently, it has been reported that inappropriate insulin release from three benign medullary-type human insulinomas was associated with disturbances in the regulation of transmembrane $Ca^{2+}$ fluxes (Flatt *et al.*, 1987*e*). Although some islet cell tumours may exhibit varying degrees of responsiveness to glucose or calcium infusion (Marks & Rose, 1981; Comi *et al.*, 1986), the present observations indicate that inappropriate $Ca^{2+}$ regulation is a common pathogenic feature underlying the inappropriate functions of certain types of insulinoma, and possibly other neoplastic cell types.

These studies were supported by grants from the Cancer Research Campaign (SP 1630), Swedish Medical Research Council (12X-562), Swedish Diabetes Association, Novo Industri AB., Swedish Hoechst and Nordic Insulin Foundation.

### References

ABRAHAMSSON, H., BERGGREN, P.-O. & RORSMAN, P. (1985). Direct measurements of increased free cytoplasmic $Ca^{2+}$ in mouse pancreatic $\beta$-cells following stimulation by hypoglycaemic sulfonylureas. *FEBS Lett.*, **190**, 21.

BEAVEN, M.A., ROGERS, J., MOORE, J.P., HESKETH, T.R., SMITH, G.A. & METCALFE, J.L. (1984). The mechanism of the calcium signal and correlation with histamine release in 2H3 cells. *J. Biol. Chem.*, **259**, 7129.

CITTADINI, A., BOSSI, L.D., DANI, A.M., CALVIELLO, G., WOLF, F. & TERRANOVA, T. (1981). Lack of effect of the $Ca^{2+}$-ionophore A23187 on tumour cells. *Biochim. Biophys. Acta*, **645**, 177.

CHICK, W.L., APPEL, M.C., WEIR, G.C. & 4 others (1980). Serially transplantable chemically induced rat islet cell tumour. *Endocrinology*, **107**, 954.

CHICK, W.L., WARREN, S., CHUTE, R.N., LIKE, A.A., LAURIS, V. & KITCHEN, K.C. (1977). A transplantable insulinoma in the rat. *Proc. Natl. Acad. Sci. USA*, **74**, 628.

COMI, R.J., GORDEN, P., DOPPMAN, J.L. & NORTON, J.A. (1986). Insulinoma. In *The Exocrine Pancreas: Biology, Pathobiology and Diseases*, Go, V.L.W. (ed) p. 745. Raven Press: New York.

CONLON, J.M., DEACON, C.F., BAILEY, C.J. & FLATT, P.R. (1986). Effects of a transplantable insulinoma upon regulatory peptide concentrations in the gastrointestinal tract of the rat. *Diabetologia*, **29**, 334.

DEVIS, G., SOMERS, G. & MALAISSE, W.J. (1977). Dynamics of calcium-induced insulin release. *Diabetologia*, **13**, 531.

DURHAM, A.C.H. & WALTON, J.M. (1982). Calcium ions and the control of proliferation in normal and cancer cells. *Biosci. Rep.*, **2**, 15.

FLATT, P.R. & BAILEY, C.J. (1981). Abnormal plasma glucose and insulin responses in heterozygous (ob/+) mice. *Diabetologia*, **3**, 573.

FLATT, P.R., BERGGREN, P.-O., GYLFE, E. & HELLMAN, B. (1980*a*). Calcium and pancreatic $\beta$-cell function: Demonstration of lanthanide-induced inhibition of insulin secretion independent of modifications in transmembrane $Ca^{2+}$ fluxes. *Endocrinology*, **107**, 1007.

FLATT, P.R., BOQUIST, L. & HELLMAN, B. (1980*b*). Calcium and pancreatic $\beta$-cell function: The mechanism of insulin secretion studied with the aid of lanthanum. *Biochem. J.*, **190**, 361.

FLATT, P.R., DESILVA, M., SWANSTON-FLATT, S.K. & MARKS, V. (1987*a*). Insulin secretion *in vivo* and *in vitro* from transplantable NEDH rat insulinoma and derived clonal RINm5F cell line. *Diabetes Res.*, **6**, 85.

FLATT, P.R., SWANSTON-FLATT, S.K., POWELL, C.J. & MARKS, V. (1987*e*). Defective regulation of insulin release and transmembrane $Ca^{2+}$ fluxes by human islet cell tumours. *Br. J. Cancer.*, **56**, 459.

FLATT, P.R., SWANSTON-FLATT, S.K., TAN, K.S. & MARKS, V. (1987*d*). Effects of cytotoxic drugs and inhibitors of insulin secretion on a serially transplantable rat insulinoma and cultured rat insulinoma cells. *Gen. Pharmac.*, **18**, 293.

FLATT, P.R., TAN, K.S., BAILEY, C.J., POWELL, C.J., SWANSTON-FLATT, S.K. & MARKS, V. (1986*b*). Effects of transplantation and resection of a radiation-induced rat insulinoma on glucose homeostasis and the endocrine pancreas. *Br. J. Cancer*, **54**, 685.

FLATT, P.R., TAN, K.S., SWANSTON-FLATT, S.K., BAILEY, C.J. & MARKS, V. (1987*c*). Defective diurnal changes of food intake, plasma glucose and insulin in rats with a transplantable islet cell tumour. *Hormone Res.*, **27**, 47.

FLATT, P.R., TAN, K.S., SWANSTON-FLATT, S.K., WEBSTER, J.D. & MARKS, V. (1987*b*). Metabolic effects and secretory properties of a radiation-induced transplantable rat insulinoma. *Comp. Biochem. Physiol.*, **87A**, 175.

GAZDAR, A.F., CHICK, W.L., OIE, H.K. & 4 others (1980). Continuous, clonal, insulin- and somatostatin-secreting cell lines established from a transplantable rat islet cell tumour. *Proc. Natl. Acad. Sci. USA*, **77**, 3519.

GRILLO, T.A.I., WHITTY, A.J., KIRKMAN, H., FOA, P.P. & KOBERNICK, S.D. (1967). Biological properties of a transplantable islet-cell tumour in the golden hamster. I. Histology and histochemistry. *Diabetes*, **16**, 409.

HANAHAN, D. (1985). Heritable formation of pancreatic $\beta$-cell tumours in transgenic mice expressing recombinant insulin/simian virus 40 oncogenes. *Nature*, **315**, 115.

HELLMAN, B. (1975). Modifying actions of calcium ionophores on insulin release. *Biochim. Biophys. Acta*, **399**, 157.

HELLMAN, B. & GYLFE, E. (1986). Calcium and the control of insulin secretion. In *Calcium and Cell Function*, Vol. VI, Cheung, W.Y. (ed) p. 253. Academic Press: New York.

HENQUIN, J.C. & MEISSNER, H.P. (1984). Significance of ionic fluxes and changes in membrane potential for stimulus-secretion coupling in pancreatic B-cells. *Experientia*, **40**, 1043.

HIRAYAMA, A., WAKABAYASHI, I., MUTO, T., WATANABE, S. & UCHIDA, S. (1979). Histological and hormonal observations on the BK virus induced pancreatic islet-cell tumours in hamsters. In *Proinsulin, Insulin and C-peptide*, Baba, S., Kaneto, T. & Yanaihara, N. (eds) p. 364. Excerpta Medica: Amsterdam.

LENZEN, S., TIEDGE, M., FLATT, P.R., BAILEY, C.J. & PANTEN, U. (1987). Defective regulation of glucokinase in rat pancreatic islet tumours. *Acta Endocrinologica*, **115**, 514.

LERNMARK, A. (1974). The preparation of, and studies on, free cell suspensions from mouse pancreatic islets. *Diabetologia*, **10**, 431.

MALAISSE, W.J., HERCHUELZ, A., DEVIS, G. & 9 others (1978*a*). Regulation of calcium fluxes and their regulatory roles in pancreatic islets. *Ann. NY. Acad. Sci.*, **307**, 562.

MALAISSE, W.J., HUTTON, J.C., SENER, A. & 4 others (1978*b*). Calcium antagonists and islet function: Effect of calcium deprivation. *J. Memb. Biol.* **38**, 193.

MARKS, V. & ROSE, F.C. (1981). *Hypoglycaemia*, 2nd edn. Blackwell Scientific Publications, Oxford.

O'HARE, M.M.T., SHAW, C., SWANSTON-FLATT, S.K., MARCELLI, M., BUCHANAN, K.D. & FLATT, P.R. (1985). Influence of a transplantable insulinoma on the pancreatic status of insulin and pancreatic polypeptide in the rat. *Diabetologia*, **28**, 157.

RALPH, R.K. (1983). Cyclic AMP, calcium and control of cell growth. *FEBS Lett.*, **161**, 1.

RINK, T.J., MONTECUCCO, C., HESKETH, T.R. & TSIEN, R.Y. (1980). Lymphocyte membrane potential assessed with fluorescent probes. *Biochim. Biophys. Acta*, **595**, 15.

RORSMAN, P. & ABRAHAMSSON, H. (1985). Cyclic AMP potentiates glucose-induced release from mouse pancreatic islets without increasing cytosolic free $Ca^{2+}$. *Acta Physiol. Scand.*, **125**, 639.

RORSMAN, P., ABRAHAMSSON, H., GYLFE, E. & HELLMAN, B. (1984). Dual effects of glucose on the cytosolic $Ca^{2+}$ activity of mouse pancreatic B-cells. *FEBS Lett.*,**170**, 196.

SWANSTON-FLATT, S.K., & FLATT, P.R. (1987). Acute and long-term effects of glucose on the function of transplantable rat insulinoma cells maintained in tissue culture. *Biomed. Res.* **8**, 215.

SWANSTON-FLATT, S.K. & FLATT, P.R. (1988*a*). Effects of amino acids, hormones and drugs on insulin release and $^{45}Ca$ uptake by transplantable rat insulinoma cells maintained in tissue culture. *Gen. Pharmac.*, **19**, 239.

SWANSTON-FLATT, S.K. & FLATT, P.R. (1988*b*). Effects of cationic modification on $^{45}Ca$ uptake and insulin release by transplantable rat insulinoma cells maintained in tissue culture. *Gen. Pharmac.*, **19**, 471.

SWIERENGA, S.H.H., WHITFIELD, J.F., BOYNTON, A.L. & 5 others (1980). Regulation of proliferation of normal and neoplastic rat liver cells by calcium and cyclic AMP. *Ann. N.Y. Acad. Sci.*, **349**, 294.

WOLLHEIM, C.B. & SHARP, G.W.G. (1981). Regulation of insulin release by calcium. *Physiol. Rev.*, **61**, 914.